# Phenotypic change of mesenchymal stem cells into smooth muscle cells regulated by dynamic cell-surface interactions on patterned arrays of ultrathin graphene oxide substrates

**DOI:** 10.1186/s12951-021-01225-4

**Published:** 2022-01-04

**Authors:** Rowoon Park, Jung Won Yoon, Jin-Ho Lee, Suck Won Hong, Jae Ho Kim

**Affiliations:** 1 Department of Cogno-Mechatronics Engineering, Pusan National University, 46241 Busan, Republic of Korea; 2 Department of Physiology, School of Medicine, Pusan National University, 50612 Yangsan, Republic of Korea; 3 Department of Biomedical Convergence Engineering, Pusan National University, 50612 Yangsan, Republic of Korea

**Keywords:** Stem cells, Self-assembly, Lithography, Tissue engineering, Smooth muscle cells

## Abstract

The topographical interface of the extracellular environment has been appreciated as a principal biophysical regulator for modulating cell functions, such as adhesion, migration, proliferation, and differentiation. Despite the existed approaches that use two-dimensional nanomaterials to provide beneficial effects, opportunities evaluating their impact on stem cells remain open to elicit unprecedented cellular responses. Herein, we report an ultrathin cell-culture platform with potential-responsive nanoscale biointerfaces for monitoring mesenchymal stem cells (MSCs). We designed an intriguing nanostructured array through self-assembly of graphene oxide sheets and subsequent lithographical patterning method to produce chemophysically defined regions. MSCs cultured on anisotropic micro/nanoscale patterned substrate were spontaneously organized in a highly ordered configuration mainly due to the cell-repellent interactions. Moreover, the spatially aligned MSCs were spontaneously differentiated into smooth muscle cells upon the specific crosstalk between cells. This work provides a robust strategy for directing stem cells and differentiation, which can be utilized as a potential cell culture platform to understand cell–substrate or cell–cell interactions, further developing tissue repair and stem cell-based therapies.

**Supplementary Information:**

The online version contains supplementary material available at 10.1186/s12951-021-01225-4.

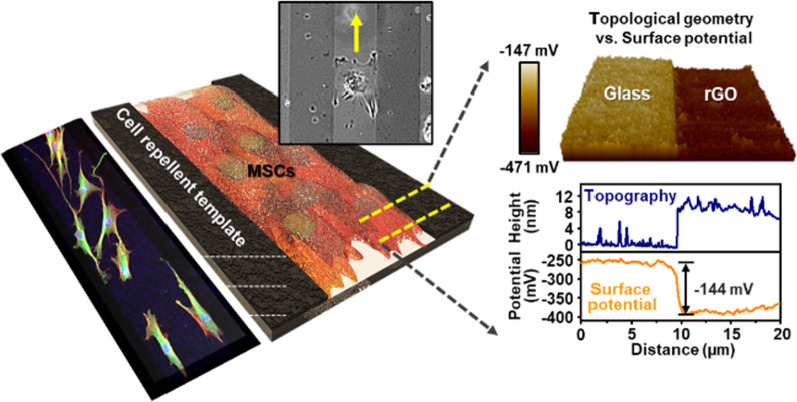

## Introduction

The ability to control cellular behavior at the cell-substrate interfaces has advanced in ex vivo biological studies through the surface chemistry and biophysical structures based on various topography, stiffness, or combinatorial properties [1–3]. The parametric biophysical cues of the substratum, produced for the extracellular matrix, play a critical role in regulating cellular behavior, including adhesion, migration, proliferation, differentiation, and apoptosis [4–6]. In particular, regarding the topographic cues that promote specific interactions at the cell-substrate interfaces, the salient features of micro/nanoscale materials have become a promising toolbox for modulating the specified cellular processes [7–9]. In this context, adhesive interactions between the cells and nanoscale interfaces have been studied extensively by facilitating unconventional lithographic or self-assembly strategies to realize the dynamic control of cells during incubation [10, 11]. For example, recently, a form of ultrathin cell-substrates, including two-dimensional (2D) nanomaterials, has provided unprecedented topographical interfaces with biologically beneficial interactions owing to their unique chemical, mechanical, electrical, and optical properties [12–15]. These materials have excellent capability as a cell culture platform because of their idiosyncratic modulatory effects, specifically on the proliferation and differentiation of stem cells [16, 17]. Therefore, it is not surprising that the interfaces with the nanoscale topographic cues of 2D nanomaterials strongly regulate the cellular behavior, altering the fate of cells, such as elongation, differentiation, and cell–cell contact and signaling. Nevertheless, there are some limitations caused by the inability to independently control the physical cues in a spatially separated configuration for stem cells to induce chemically different contact adhesion, even though topographical cues on the nanoscale 2D materials have been suggested as a powerful cell substrate to control cellular behavior [18–20]. Moreover, the possible cell-repellent effects associated with the micro/nanostructured features of 2D materials in directing cell migration and aligned cell organization have received little attention.

Here, we report acute potential-responsive nanoscale biointerfaces to regulate and monitor stem cells through the self-assembly of a 2D nanomaterial (i.e., graphene oxide, GO) and subsequent lithographical method to produce a spatially discrete patterned surface. The prepared ultrathin cell-culture platform could control cell migration triggered by focal-adhesion formation within the alternately confined geometry. Recent progress of synthetic manner for 2D materials expanded their capability for use in cell culture substrate to enhance cellular behaviors because of excellent biocompatibility, biodegradability, or drug-loading functionality [21–23]. Thus, physicochemically defined nanostructured films could be manipulated to direct the living cells and to promote spontaneous cell adhesion, migration, alignment, and differentiation. In this context, the progress in research was only lied in the film fabrication strategy using individually well-dispersed colloidal nanosheets in creating nanostructured films [24]. On this, the GO solution is one of the most easily accessible 2D materials to generate uniform films with a high yield, compared to other 2D materials. Therefore, many of the biological interfaces based on GO films has been developed to observe acute cell responsive interfaces and guided cell migration into a desired shape or area within a delicately defined physicochemical environment [25, 26]. Moreover, the synergistic potential of the topography-mediated differentiation could be revealed by rendering a critical cell-repellent environment for a specific stem cell [27, 28]. As a model system for this experiment, we selected easy-accessible mesenchymal stem cells (MSCs), which are considered a promising candidate for tissue regeneration in regenerative medicine because they can be isolated from most organs and tissues [29]. MSCs possess rapid self-renewal capacity and versatile potential to differentiate into various cell types, such as adipocytes, osteoblasts, chondrocytes, and smooth muscle cells [30, 31]. In particular, the differentiation of MSCs into smooth muscle cells could be stimulated by various extracellular agonists, including transforming growth factor (TGF-β1), sphingosylphosphorylcholine, and prostaglandin F2α [32–34]. On the other hand, it is unclear if the external stimuli explicitly affect the differentiation of MSCs to smooth muscle cells (SMCs), but accumulating evidence suggests that the control of nanoscale topological features affects the morphology and differentiation of MSCs to osteogenic or neurogenic cells [35, 36]. In this study, we focused on the architecting of the ultrathin physicochemically defined cell-substrate and explored the related interactive responses of MSCs with tightly balanced cellular behaviors between self-restrained migration and differentiation into SMCs toward a highly aligned and stretched configuration. To accomplish this, the MSCs were cultured on the alternately patterned arrays of ultrathin reduced GO (rGO) films. rGO provided unique structural support and regulated the cell fate in the surrounding local geometries by expressing newly developed cellular behaviors with the systematic extrinsic factor signals. This robust yet straightforward strategy represents an important application of the rGO-based patterned substrate to influence the differentiation of stem cells towards correct tissue homeostasis [37].

## Results and discussion

### Designing of ultrathin patterned substrate with a topographically/chemically defined region

**Fig. 1:**
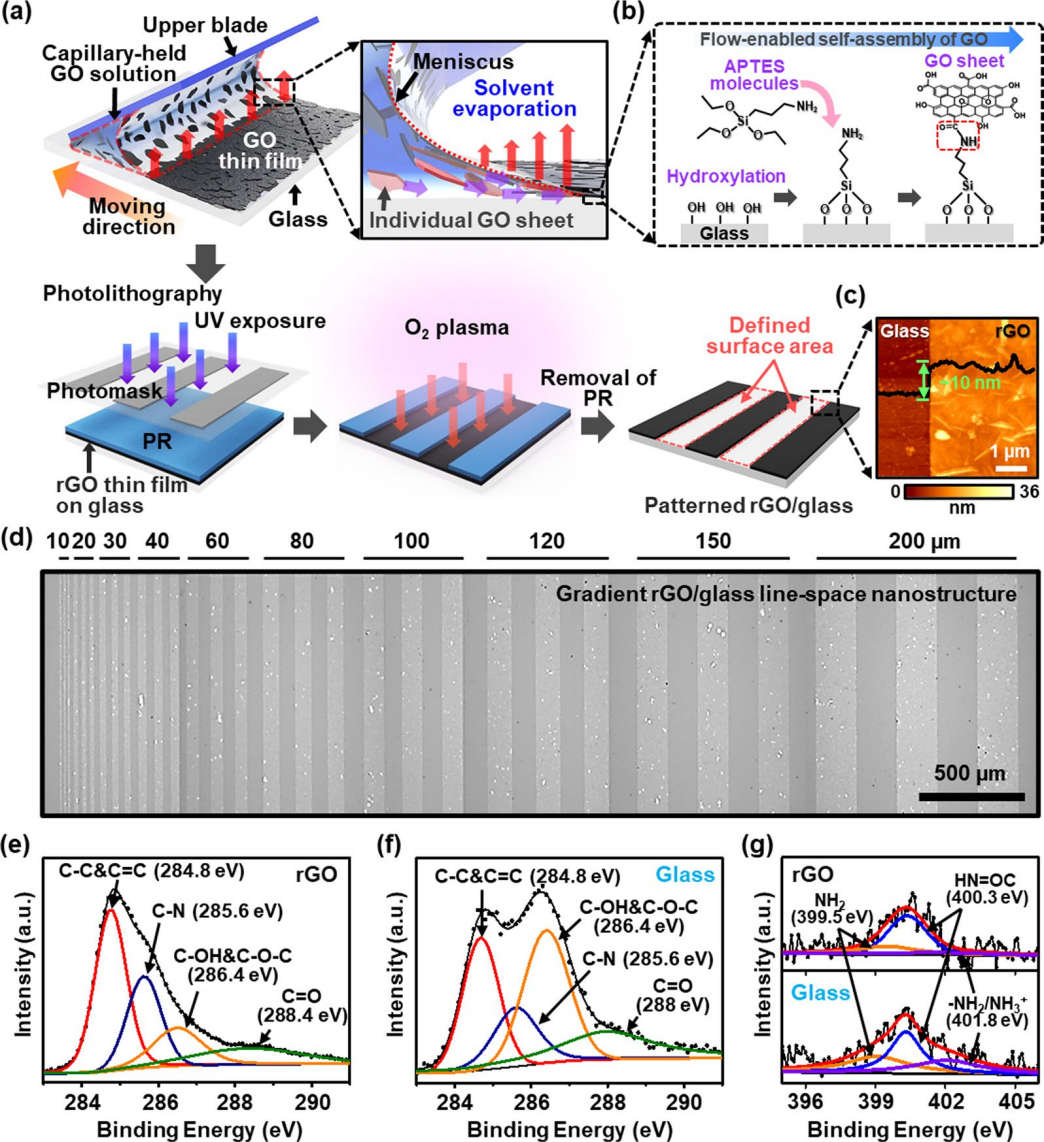
**a** Schematic illustration presenting the fabrication process of the patterned rGO substrate with a topographically/chemically defined region. **b** Schematic steps for the surface modification of glass substrate through a hydroxylation process using piranha solution and sequential silanization by 3-aminipropyltriethoxysilane (APTES) treatment. **c** Representative atomic force microscopy image of the boundary region with the height profile; the scan size was 5 × 5 µm^2^. **d** Optical micrograph of alternately patterned rGO ultrathin films with defined widths ranging from 10 to 200 μm. **e**, **f** The C1s XPS spectra collected on the rGO region and exposed glass region. **g** The N1s XPS spectra of rGO and glass surface

Figure 1a presents a schematic illustration of the sequential process to produce an rGO-based patterned substrate with a topographically defined region. At the first step, abundant surface hydroxyl groups were introduced to a flat glass substrate through a hydroxylation process using piranha solution, and a self-assembled monolayer (3-aminopropyltriethoxysilane, ATPES) with terminated amine groups (–NH_2_) was subsequently formed onto the hydroxylated glass surface (Fig. 1b). A well-dispersed GO suspension (c = 2 mg ml^−1^) was injected in a restricted geometry composed of the upper blade and lower substrate (i.e., APTES-modified glass substrate) positioned at a 30° angle. The capillary-held GO meniscus was naturally formed at the bridge of the fixed gap between the upper blade and the substrate (~100 μm). In this unique geometry, the motor-driven translation stage connected to the lower substrate was traveled back-and-forth repetitively along the programmed route at a constant velocity of 5 mm s^−1^. As the trapped GO meniscus passes over the substrate, the capillary-induced flow tends to migrate to the GO sheets toward the contact line of the meniscus, and the individual GO sheets are deposited uniformly after consecutive solvent evaporation at the contact line of the GO meniscus (inset image in Fig. 1a and see Additional file 2: Movie S1) [38]. During this process, the planar stacking GO sheets, containing the carboxylic (–COOH) group at the edges and basal planes, were bonded covalently with the terminal amino groups of the APTES monolayer on a glass substrate [39]. Next, the thermal annealing step was performed at 200 °C for 8 h to strengthen the CO–NH linkage in the GO-APTES coupling by eliminating the oxygen groups of the GO basal plane. At this stage, GO was transformed to the rGO form [40]. The alternately patterned surface area was defined by conventional photolithography on the rGO film/glass substrate using a mask-aligner with a photomask of micron line/space pattern in a gradient configuration. The exposed GO regions (i.e., unprotected by photoresist) were removed completely using O_2_ plasma. Finally, the photoresist was stripped using a resist remover, leaving behind the patterned rGO films on the glass substrate. The boundary between the rGO and glass was measured by atomic force microscopy, which revealed a sharp contrast at the edge as presented in Fig. 1c. The thickness and surface roughness were also extracted from the cross-sectional height profiles, in which the thickness of the rGO stripe was identified with the ultrathin features of ~10 nm-thick over the patterned surface areas with relatively low surface roughness of ~2.5–3.2 nm (root-mean-square, RMS value). In particular, the rGO stripes (i.e., line/space pattern) produced via flow-enabled self-assembly and the lithographic pattern-transfer process were yielded an ultrathin film of stacked configuration that topologically separated on the planar structure in a single substrate. Figure 1d shows a representative optical micrograph of a gradient and periodically patterned rGO surface with a topographically defined as line-width of 10 to 200 μm, where the clear contrast of the stripes with different local densities of the micropatterned rGO films (bright) and glass (dark) was appeared.

This design is critical in our experimental concept because the trend in the alignment of the cultured MSCs can be affected by the microenvironment provided and the intrinsic size of the individual cells in differentiation, as previously reported [41]. Therefore, this topologically gradient patterned surface as a cell-culture substrate was perfectly fit for the systematic studies in the present experimental scheme under in situ observation to uncover the focal adhesion of the cells and relevant migration responses. Prior to culture the stem cells on the rGO patterned substrate, we thoroughly examined the structural characteristics of the rGO films on a glass substrate because the physicochemical property of the substratum is one of the key parameters to control the stem cell behaviors. First, the collected Raman spectrum showed two main peaks, which were assigned to the D band (1328 cm^−1^) and G band (1569 cm^−1^) as presented in Additional file 1: Figure S1. The D band denotes the sp^3^ structural defects in the rGO basal plane. In contrast, the G band is related to the restoration of the sp^2^-bonded carbon lattice by eliminating oxygen-functional groups. In the graph, the G band showed higher intensity than that of the D band, suggesting that the reduction to GO proceeded through the structural restoration of the sp^2^-bonded carbon lattice due to the dissociation of the oxygen in the GO domain during annealing [42]. In addition, the optical properties of the transmittance were evaluated by UV–Vis spectroscopy over the wavelength range, 400 to 800 nm, for the rGO film and patterned rGO thin film as shown in Additional file 1: Figure S2. The patterned rGO thin film exhibited a high optical transmittance above 92% at 550 nm through the O_2_ plasma etched opening, compared to the rGO thin film on a glass substrate (T = 83.7% at 550 nm wavelength). This clear view field is beneficial to the observation of cell migration.

To identify the details of surface chemistry generated by the sequential fabrication process, we examined the rGO/glass regions quantitatively using X-ray photoelectron spectroscopy (XPS), as summarized in Fig. 1e–g. As designed, the manipulated layered surface can be considered rGO/APTES/glass and O_2_ plasma-exposed APTES/glass in the molecular definition. For the rGO/APTES/glass region (Fig. 1e), the C1s spectra identified a series of C–C, C–N, C–O, and COOR species at 284.8, 285.6, 286.4 eV, and 288.4 eV, respectively; this result is consistent with the chemical structure model of rGO [43]. The distinct component area of the C–N peak at BE = 285.6 eV originated from the internal bonding of the carboxylic group in the rGO basal plane, the amine groups, and the presence of the unbound terminal groups of APTES. A major component of the C–N peak similarly appeared in the APTES/glass region that could be attributed to the oxidation reaction of amine head groups unbound with GO sheets on the glass surface during the O_2_ plasma process, but the slightly increased C-O peak was detected as a result of the destruction of amine groups and the subsequent substitution of hydroxyl groups [44]. Indeed, the impact of O_2_ plasma was evaluated by the direct comparison to the survey of C1s spectrum for the originally provided APTES-modified glass surface, revealing the C-N component peak (Additional file 1: Fig. S3a). On the other hand, for the N1s XPS spectra, HNO=C at BE = 400.3 eV was dominant in the rGO region, confirming the existence of NH_2_ terminal group (BE = 399.5 eV) and weak hydrogen bond/protonated amines (–NH_2_/NH_3_^+^, BE = 401.8 eV) as shown in Fig. 1g. The major component peak represents an amide formation (HN=OC) on the APTES/glass region after the O_2_ plasma process. Hence, the APTES/glass regions configured with rGO/APTES/glass were only optimized with copious free amines before the O_2_ plasma process (Additional file 1: Fig. S3b). This supports the interpretation of the C1s spectrum associated with the amide group. For more information, as summarized in Table S2, the intensity ratios of the oxygen-containing groups to the graphite component were fully evaluated to quantify the effective charge compensation in each sample. Remarkably, the O_2_ plasma-exposed glass regions contain a higher hydroxyl carbon content than the photoresist-blocked rGO and as-prepared APTES-modified glass regions, which suggests that the silanolated surfaces are saturated with a high degree of oxidation. Moreover, the increased level of carboxyl carbon content (HNC=O) also indicates the silanol and oxidized amine terminated surface. Finally, physically and chemically defined cell culture substrates were characterized with rGO/glass arrays of gradient micro-widths and equal heights to study how cells form adhesions and spread through them.

### MSC culture on the chemophysically defined biointerfaces

**Fig. 2:**
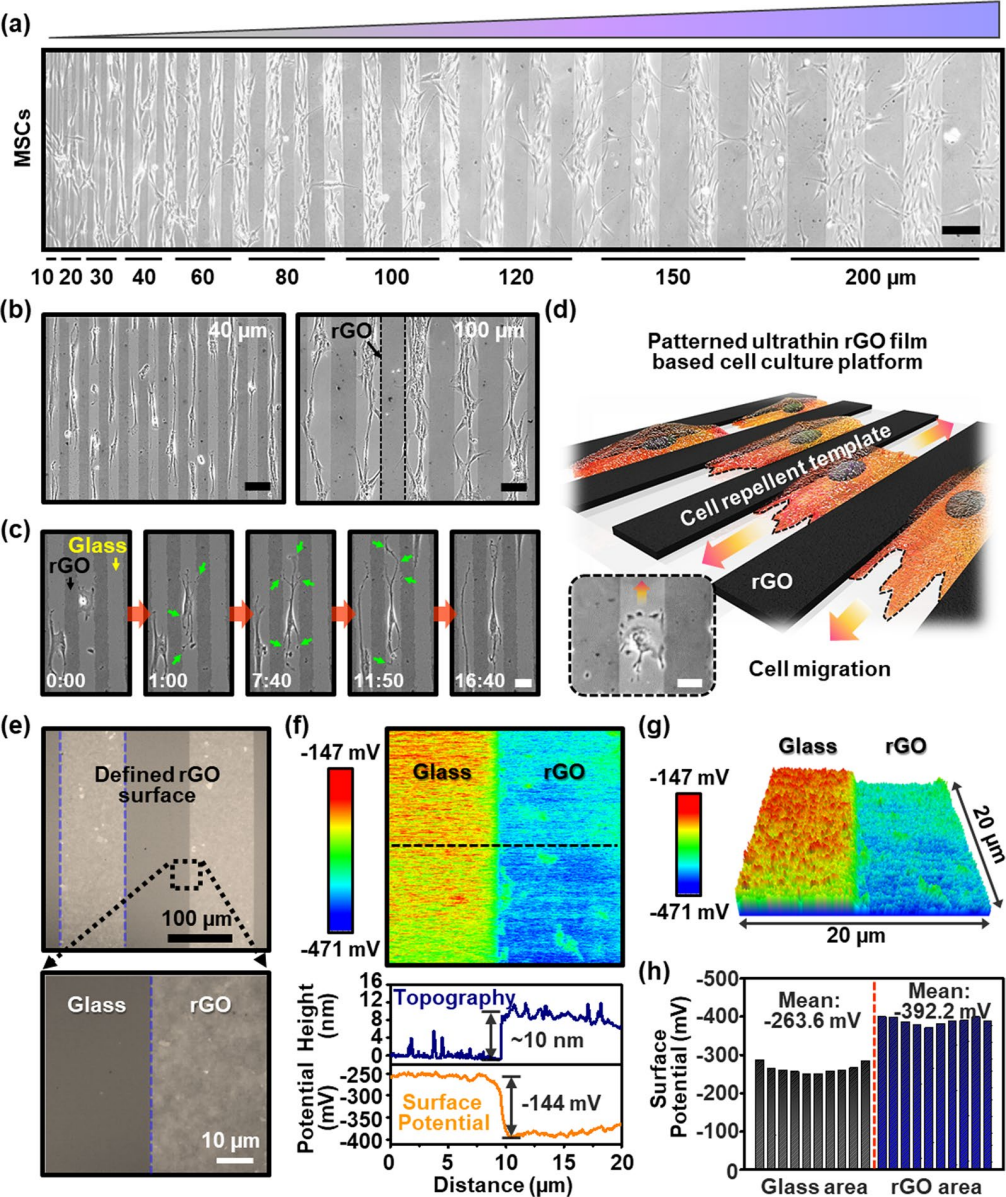
**a** Optical micrographs of the MSCs cultured on the gradient pattern spacing of glass/rGO substrate, scale bar: 200 μm. **b** Optical micrographs of MSCs on the anisotropic cell-substrate of different pattern spacing of 40 and 100 μm, respectively, scale bar: 100 μm. **c** Real-time observations of cell migration on pattern spacing of 40 μm; captured time intervals (min s^−1^): 0:00/1:00/7:40/11:50/16:40, scale bar: 40 μm. **d** Conceptual images of engineered cellular behaviors through the rGO-based patterned substrate with the topographically/chemically defined region; the inset image represents the migrating MSC while recognizing the glass surface between rGO patterns, scale bar: 50 μm. **e** OM image of the alternately patterned rGO films; the inset image indicates a boundary between the rGO and glass surface. **f** KPFM mapping image of the surface potential difference between rGO and glass surface; the scan size is 20 × 20 µm^2^. **g** 3D KPFM images of micro-patterned glass/rGO substrates with regions separated by potential differences. **h** Potential distribution mapping at each glass and rGO region; the mean charge potential was extracted at a size of 4 × 4 µm^2^

The selective surface recognition and cell-specific responses to the patterned microenvironment were examined by culturing MSCs separately on the prepared substrates: glass, rGO, and the alternately patterned rGO stripes on the glass. No significant differences in cell adhesion and proliferation were observed on the glass and rGO film/glass without patterns, as shown in Additional file 1: Fig. S4a and b. However, unexpected cell responses on the alternately patterned substrates were exerted as presented in Fig. 2a. In the anisotropic patterned cell substrate, a highly aligned configuration of MSCs was evaluated overall in the entire area. Specifically, the morphological evolutions of MSCs was obviously appeared by the extraneous surface-initiated cues in an aligned features with gradient cell densities on the glass regions adjacent to the rGO stripes. With a careful observations presented in Fig. 2b and Additional file 1: Fig. S5, the highly aligned and bundled MSCs were densely connected by clustering in the range of 100 μm or more on the glass stripes, whereas the cells were distributed as a single by end-to-end cell configuration within the patterned area of the 40 μm. Furthermore, an effective movement of the seeded MSCs on the glass/rGO substrate was monitored in real-time and traced for the direct assessment of cell behavior in a controlled environment as captured in Fig. 2c (see Additional files 3, 4: Movies and Additional file 1: Fig. S6). Notably, the individual single motile cells resided only on the glass stripes and interconnected with each other, guided by the adjacent edges of the rGO stripes. From a macroscopic perspective, most of the cells were polarized, and they stretched their morphologies biaxially along with the parallel directions of the patterned glass stripes by actively sensing the exposed surfaces. This observation is critically important because the morphological changes to stem cells caused by contact guidance can alter their cellular responses associated with the differentiation, in which the alignment of the cells is essential. In mechanobiology, control of the dynamic organization of large protein complexes between actin filaments and integrins (i.e., focal adhesions) can be one of the main parameters to examine the physicochemical cues for the cell functions, related to the intercellular tension and stress in the actin cytoskeleton [45–47]. Within a given unique microenvironment, Fig. 2d depicts a main conceptual schematic diagram of cell alignment and elongation, that is, the surface-mediated cell adhesion and migration of the MSCs.

In fact, at the initial stages of the experiment, we hypothesized that the isometric rGO/glass stripes might induce morphological reconfiguration of the stem cells during culture, which could be effective interfacial guidance on the discriminated cellular behavior, such as alignment, orientation, and cell-cell communication [48–51]. Recent studies reported that the cells cultured on graphene-activated substrates exert favorable cell–substrate interactions through the development of focal adhesion sites [52–54]. Moreover, in our previous work [13], a favorable effect on the nanoscale surface topography of the patterned rGO film was fully evaluated to study beneficial interfacial interactions of living cells such as fibroblasts, myoblasts, and neuronal cells, in which we found that the nanoscale topography of the patterned rGO surfaces enhanced cellular behaviors such as adhesion and differentiation. However, in the present study, a similar but different approach was set with the chemically functionalized conditions in the same thickness range of the patterned rGO films (i.e., ~10 nm). Surprisingly, unexpected cell responses were detected by representing repellent interactions with the patterned rGO films. This suggests that the hetero-electrostatic interactions or inherent nanoscale topographies of graphene surface can be synergistic with the help of patterning strategies for the cell behavior rather than the fully covered graphene thin-film substrate by promoting migration and encouraging cell–cell communication [21]. Based on these firmly established assessments of cell cultures, we assumed that a delicate control of the anisotropic microenvironments from the favorable cues from the ultrathin patterned rGO films spatially provide distinctive cell interactions on each surface region. However, in this experimental result, graphene-repellent behaviors of the MSCs contrast with the existed contact guidance of graphene surface for living cell assembly [17, 49]. This implies the predictable design of the tissue scaffold for the specified stem cells (e.g., MSC) is difficult and controversial within the limited understanding involved in the precise mechanisms of the cell-repellent behaviors, responsible for the acute cellular responses on each provided microenvironment [55]. Therefore, we investigated the levels of the surface-potential distributions on the topological culture environment to understand one possible consideration of the heterogeneous electrostatic interactions in patterned rGO/glass arrays that could trigger unprecedented cellular responses.

Figure 2e presents magnified optical micrographs for the defined surface area of rGO/glass stripes with 100 μm line-intervals. As seen in this configuration, the boundary between the rGO and glass was geometrically isolated with sharp contrast. In this structure, to scrutinize the prepared biophysical interfaces accurately the potential distribution of the surface charge across the glass/rGO stripes was evaluated by kelvin probe force microscopy (KPFM) with spatial mapping data, as visualized in Fig. 2f. It should be acknowledged that the surface charge differences on the glass/rGO regions were clearly examined with a certain potential level (Fig. 2g). For example, the height profile for the surface topography of the area showed a higher magnitude in the rGO regions (~10 nm), but the corresponding distribution of surface potential was ~144 mV more negative than the value on the glass region (bottom panel in Fig. 2f and Additional file 1: Fig. S7). The statistical data distributed over each segmented region (i.e., glass and rGO surface) indicates obviously separated and relatively uniform locational average charge potential levels of -263.6 mV and -392.2 mV on the glass and rGO region, respectively (Fig. 2h and Additional file 1: Fig. S8).

**Fig. 3:**
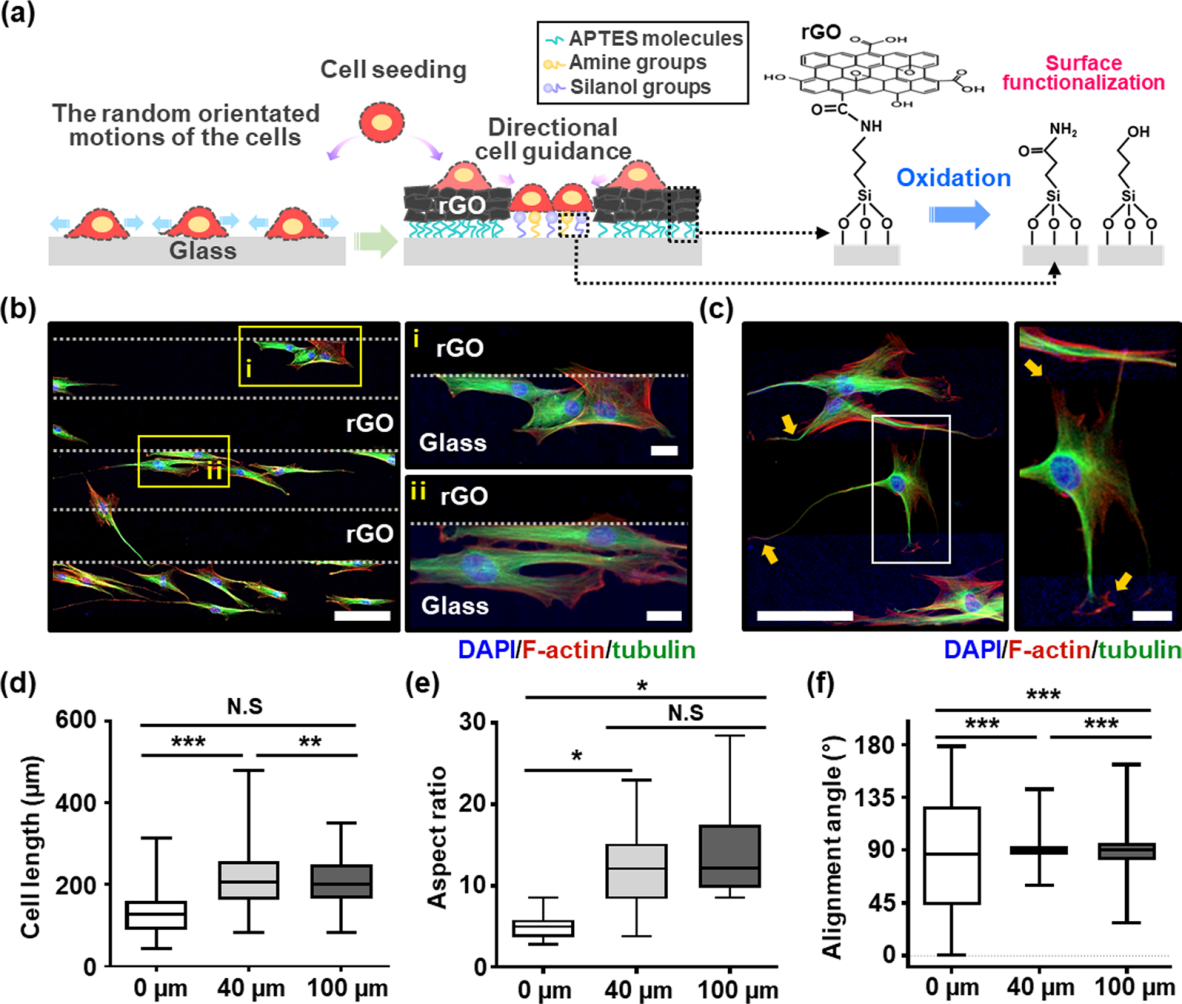
**a** Schematic diagram illustrating MSCs adhesion and migration on anisotropic patterned rGO and glass regions with spatial topographies and surface potentials; the layered rGO sheets in the rGO region impart geometric features, and the potential difference with the rGO region is generated by the coexistence of amine and silanol groups in the glass region. **b**, **c** Fluorescence micrographs for the expression of F-actin (stress fiber, red), tubulin (microtubule, green), DAPI (nuclei, blue) in MSCs, scale bar: 100 μm (left in **b**, **c**) and 20 μm (right in **b**, **c**). Quantitative analysis of MSC morphologies, such as the cell length (**d**), aspect ratio (**e**), and alignment angle (**f**) on 0 μm (unpatterned glass, n = 124), 40 μm (n = 137), and 100 μm (n = 104), of pattern spacing, respectively (*p < 0.05, **p < 0.01, ***p < 0.001 versus 0 μm group)

So far, such regional physicochemical differences in the cell culture substrate that is produced by the combining construction of the nanoscale materials and chemical modifications were well suited to cell culture analysis, and the critical surface properties were precisely evaluated by XPS and KPFM analysis for the patterned rGO/glass array. Figure 3a schematically describes the possible principle of the surface-mediated assembly of the MSCs based on the directional cell guidance effect and preferential adhesions. In the sequential process to prepare the patterned rGO/glass array, a strong negative charged surface was maintained at the rGO regions (i.e., ~ 392.2 mV) due to oxygen-related functional groups such as hydroxyl, carbonyl, and carboxyl groups. In contrast, the glass surface was subtly complexed with oxidized amide and silanol groups when exposed to the mild O_2_ plasma process. On the copious silanol-functionalized glass surfaces, the oxidized amide groups mainly contribute to mediate a surface potential level, introducing a relatively less negatively charged surface (i.e., ~ 263.3 mV) compared to the rGO regions, which leads to a fundamental heterogeneity of the patterned surfaces. Hence, the heterogeneity in each separated potential level provided selective recognition sites to the MSCs and guide them to reside on the favorable silanol and amine functioned surfaces, which is obviously in contrast to the case of the random orientated cell configurations on the unpatterned planar cell substrate [56, 57]. Thus, it can be inferred that, in our experimental system, the delicate interactions of the cells on the patterned substrates contributed more importantly to controlling cell behavior than other parameters due to the anisotropic surface potential difference.

Consequentially, the morphological features of MSCs adhered were observed by labeling the cytoplasmic microtubule (i.e., tubulin) and stress fiber (i.e., F-actin) by immunofluorescent staining and merging the images. The cells exhibited the highly confined cell alignment with enhanced focal adhesions (Fig. 3b). In particular, the microtubules were organized only within the glass stripes by the presence of the cell-repellent rGO interfaces. Clear evidence was presented by highly magnified micrographs in the marked area of (i) and (ii) in Fig. 3b. On the 100 μm patterns, multiple cells were connected by contacting each other, and the actin filaments were fully stretched only on top of the glass stripes as contact guidance to confine the MSCs to prevent crossover of the rGO regions (Fig. 3c and Additional file 1: Fig. S9). As a result, the restricted cell migration and attachment were analyzed statistically with characteristic features, as summarized in Fig. 3d–f. We focused on the cell capture capabilities for the representative pattern spacing (i.e., 40 and 100 μm), which verified the morphological evolutions of the MSCs with highly aligned cell colonies. Note that the effective local guidance for MSCs was more significant than the control culture on a flat glass substrate (i.e., absence of patterned rGO arrays), which displayed a universal random growth without a preferential orientation (Additional file 1: Fig. S10). Compared to the randomly seeded MSCs, the increased cell length was observed on the 40 μm pattern spacing substrate up to ~ 500 μm with self-confined geometrical factors upon organization (Fig. 3d and Additional file 1: Fig. S11). The aspect ratio, dividing the length by the width of each cell, also increased significantly in both 40 and 100 μm pattern spacing, following the directional guidance of the glass stripes (Fig. 3e). Comparable to this data set, the orientation of the cell alignment was generally close to 90° with a narrow distribution on the prepared patterned substrates (Fig. 3f); 90° denotes a parallel position on the axis of the stripes, and 0° represents perpendicular alignment (Additional file 1: Fig. S12). A more extensive study of the specific features on the cell attachment and selective arrangements were surveyed in detail, as shown in Figures S13 and S14, in which a cross-patterned glass surrounded by ultrathin rGO film was used to observe the cell migration toward the active target area (i.e., only exposed glass region) in real-time. Most MSCs migrated to a cross-shaped glass region (Additional file 1: Fig. S13) and stretched their morphologies within the restricted area or aligned sharply at the circular borderline between the glass and enclosed rGO film stretching the F-actin fibers (Additional file 1: Fig. S14). This set of results also demonstrated that MSCs prefer to bind on the favorable glass surface, directly compared to the rGO film surface.

### Spontaneous surface-induced differentiation of MSCs to SMCs

**Fig. 4:**
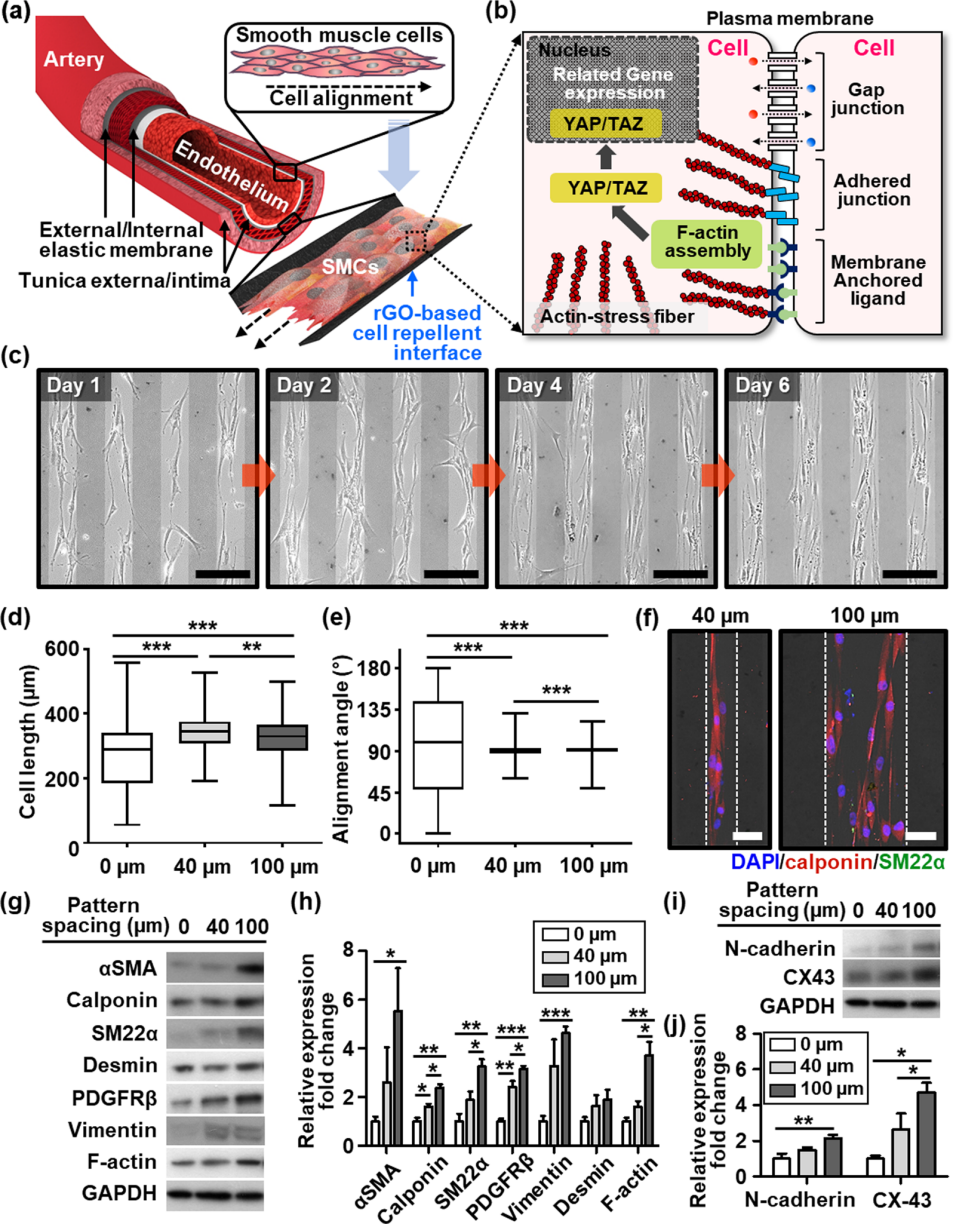
**a** A concept description of a biomimetic microenvironment of the arterial wall on the function of the cell repellent patterned cell culture substrate, in which the circumferentially organized SMCs are surrounded by the elastic membrane. **b** The schematic illustration represents intracellular signaling promoted by cell-to-cell connections. **c** Optical micrographs of quiescent MSCs cultured in serum-free media on 100 μm of pattern spacing for six days, scale bars: 200 μm. Quantitative analysis of quiescent MSCs morphologies cell length (**d**) and alignment (**e**) angle on 0 μm (unpatterned glass, n = 112), and pattern spacing of 40 μm (n = 116) and 100 μm (n = 112), respectively. **f** Zoom-in fluorescence micrographs for the expression of SMC marker, calponin (red), SM22α (green), of the highly aligned MSCs treated by TGF-β1 for six days of culture, scale bars: 50 μm. Western blot analysis to compare the expression of smooth muscle cell markers (**g**) and cell-to-cell connection markers (**i**) in MSCs on each substrate at day six and GAPDH used as the loading control. **h**, **j** Graphs for expression level quantification were normalized to GAPDH as the fold increase compared to the control (0 μm). Data are shown as the mean ± SD (n = 3). (*p < 0.05, **p < 0.01, ***p < 0.001 versus 0 μm group)

From a stem cell functionalization point of view, our presented work could be effectively utilized to constitute a form of sheets of stranded SMCs in a highly aligned configuration. For example, as conceptually presented in Fig. 4a, the diameter of blood vessels is controlled by the highly oriented SMCs to regulate blood flow and pressure. Generally in the organ parts, SMCs are longitudinally or circumferentially organized around the internal tissue layers of the visceral organs that are mainly responsible for the proper contraction and relaxation of the organs in gastrointestinal, cardiovascular, urinary, and reproductive systems [58]. Therefore, the control of cellular orientation could be one of the important issues in engineering stem cell application, especially for SMCs. Because the cell sheet based on the aligned arrays of SMCs may endow a therapeutic function for implanted tissues with controlled cell morphology (i.e., aligned array), the progressive goal of cell sheet engineering may be to create vascularized tissue-like structures. Moreover, the layers of cells can be stratified, composed of more than one cell type, in a patterned manner [59]. As presented earlier, the MSCs rely on anisotropic contact guidance, exhibiting an elongated cytoskeletal structure located mainly on the specified micropatterns. Notably, spontaneous “cell-to-cell contact” can be inevitable in a confined microenvironment (Fig. 4b), resulting in cytoskeletal remodeling caused by F-actin assembly [60–63]. This restricted condition may cause cell differentiation by activating YAP/TAZ, which is the key transcription factor of the Hippo signaling pathway, as illustrated in Fig. 4b [64, 65]. With the encouraging results thus far and accumulating evidence that MSCs can differentiate into SMCs [28, 66, 67], the effect of defined guidance cues from the rGO stripe pattern was maximized by synchronizing MSC differentiation into SMCs as a proof-of-concept model (Additional file 1: Fig. S15).

In addition, the effects of the substrate as physicochemical guidance for the differentiation of MSCs into SMCs were examined by culturing the MSCs on two different rGO pattern spacings (i.e., 40 and 100 μm) and flat glass substrates under serum-free conditions or with a TGF-β1 treatment as an agonist inducing the differentiation of MSCs to SMCs. The serum-deprived MSCs were aligned directionally on the anisotropic patterned cell substrate until day-6 of incubation to induce the gradual development of the stress fibers (Fig. 4c and Additional file 1: Fig. S16). The MSCs were arranged strictly in a unidirectional alignment by the anisotropic chemo-physical cues, where the markedly aligned arrays of the quiescent MSCs could be observed in the overlapped configuration after the six days of culture. As shown in Fig. 4d, the cells exhibited the stretched phenotypes on the pattern spacing of 40 and 100 μm, unlike the control groups (i.e., cultured on unpatterned substrates). By initially driven acute contact guidance, the quiescent MSCs expressed spontaneous morphological features with a highly aligned configuration (Fig. 4e and Additional file 1: Fig. S17), maintaining cellular integrity during cell cultivation. The bundling of MSCs with cell-to-cell contacts may reflect the morphological characteristics of the SMCs. Furthermore, the directional guidance with unidirectional alignment increased the cell-to-cell contact area between the cells dramatically during the TGF-β1-induced differentiation of MSCs to SMCs on the patterned cell substrate (Fig. 4f). Thus, cell-to-cell contacts could be induced by increasing the pattern spacing with an optimal range of 40-100 μm (Additional file 1: Fig. S18a). Accordingly, the cell alignment and orientation were unaffected by the TGF-β1 treatment during differentiation to SMCs (Additional file 1: Fig. S18b). The relevant differentiation capacity was uniformly retained, which was visualized as calponin and SM22α marker expression in Additional file 1: Fig. S18c.

The influence of the microenvironment on differentiation of the quiescent MSCs to SMCs was examined by measuring the expression levels of myogenic markers by western blot analysis, as shown in Fig. 4g–j. Intriguingly, the expression of SMC markers, including α-smooth muscle actin (α-SMA), calponin, and SM22α, was more increased at the 100 μm pattern spacing than the other cases (i.e., unpatterned or 40 μm pattern spacing), suggesting that broader cell-clustering accelerates the differentiation of MSCs to SMCs, as shown in Fig. 4g, h. Furthermore, smooth muscle tissue consists of multiple SMCs connected through connexins stimulated in a synchronous pattern. The transmembrane protein (i.e., N-cadherin) mediates cell-cell adhesion in multiple tissues [62, 63, 68, 69]. As shown in Fig. 4i and j, the expression of N-cadherin and connexin 43 also increased in the 100 μm pattern spacing. To quantify the expression of SMC-specific markers, flow cytometry analysis was performed. As presented in Additional file 1: Fig. S19, the MSCs cultured at a pattern spacing of 100 μm exhibited increased expression of calponin and SM22α compared to the MSCs cultured at a pattern spacing of 0 μm. Although the aligned MSCs represented a lower expression of SM22α than SMCs induced by TGF-β1, the expression levels of calponin were almost similar in these cells. Furthermore, MSC (control), TGF-β1-induced SMCs, and MSC cultured at pattern spacings of 0 and 100 μm were subjected to collagen contraction assay to determine the contractile activities of cells (Additional file 1: Fig. S20). MSCs cultured at the pattern spacing of 100 μm showed markedly reduced collagen gel area compared to the MSCs cultured at the 0 μm pattern spacing, but had a size similar to that of collagen gel lattices containing SMCs induced by TGF-β1. Therefore, these results suggest that anisotropic alignment induces differentiation of MSCs to SMCs which can contract the collagen gel lattices.

The cell shape can influence Hippo signaling and mechanical tension transmitted through cell-cell junctions and cell-matrix adhesions [64, 65]. Therefore, the expression of YAP and TAZ was determined by Western blotting. As shown in Additional file 1: Fig. S21, TAZ expression in the 40 μm pattern spacing increased more than that in the unpatterned substrate and was further augmented in 100 μm pattern spacing consistent with an increase in α-SMA expression. In contrast, YAP was expressed at relatively low levels with no significant differences. TAZ is likely to have possible implications in the myogenic differentiation of MSCs on a chemophysically defined cell substrate because it plays a vital role in regulating myogenesis and cytoskeleton organization promoting the phenotype of quiescent SMC [67, 70]. By facilitating the arrays of ultrathin rGO patterns on a glass substrate, the present study demonstrated that the cell-culture platform, with anisotropic features in the surface structure, induced phenotypic conversion by modulating the adhesion, migration, and differentiation of human MSCs.

## Conclusions

A simple strategy was developed to produce alternately patterned rGO arrays on a glass substrate for an anisotropic cell-culture platform, which consisted of ultrathin topological features separated by discrete surface potentials using the multiscale self-assembly and conventional photolithography. The prepared cell substrate was chemo-physically defined by precisely measuring the spatial topographies and surface potentials using a KPFM technique. The unexpected subtle interfacial interaction of MSCs on such a unique microenvironment was studied extensively to unravel the manipulated cellular responses and harmonize them into controlled cell assemblies in a highly aligned configuration, imposing appropriate functions for the organized MSCs. The spatially aligned stem cells were governed by specific crosstalk between the cells, such as cell-to-cell contacts, to regulate the cell fates and differentiation. The major protocols described in the present work are easily adaptable to large-scale manufacturing, enabling control of the stem cell onto the highly ordered organization in a controlled manner. Furthermore, based on the versatility of the pattern size and geometry, which relies on the structural shape of a mask during photolithography, the cell-repellent rGO based pattern can be extended easily to investigate the optimal biophysical and physicochemical cues of the substratum to enhance the cell type- and patient-specific stem cell-based therapies. The cell-repellent-based biomimetic interfaces in the patterned arrays of ultrathin nanoscale films are currently under investigation toward versatile uses of a cellular responsive ultrathin scaffold onto specific cell types for cell separation. Overall, this work will advance stem cell differentiation by providing a new strategy to design and develop cell substrates and help understand the fundamental interactions between the cell and substrate interface, thereby driving more effective stem cell-based therapies.

## Materials and methods

### Materials

α-minimum essential medium (12000022), fetal bovine serum (A12617), and trypsin-EDTA (25300054) were purchased from Thermo Fisher Scientific (Waltham, MA). Hank’s balanced salts solution (H4891), thiazolyl blue tetrazolium bromide (MTT, M5655), and bovine serum albumin (A8806) were obtained from Sigma-Aldrich (St. Louis, MO). TGF-β1 (240-B-002) was supplied by Peprotech (Rocky Hill, NJ). Table S1 lists the antibodies used in this study.

### Surface modification of the glass substrate

Before fabricating a uniform GO thin film on the glass substrate via the FESA process, the glass substrate was hydroxylated using piranha solution (mixture of sulfuric acid and hydrogen peroxide in a 2:1 volume ratio) and then washed with deionized water. After hydroxylation of the glass surface, the glass substrate was immersed immediately into a prepared 3-aminopropyltriethoxysilane solution (APTES, 99% purity, Sigma, St. Louis, MO, USA) at a volume ratio of 1:5 in a mixture of deionized water and acetone for 2 h at room temperature. The glass surface was modified with the terminal amine groups of the APTES self-assembled monolayer. The silane residue (unbound APTES molecule) was washed off with acetone and blown with N_2_ gas.

### The fabrication of the rGO-based patterned substrate

The GO suspension was produced using the modified Hummer method, as described [71]. GO sheets were dispersed in deionized water to a concentration of 2 mg ml^−1^. Subsequently, 10 µl of an aqueous GO suspension was injected in a confined geometry composed of an upper blade and lower substrate (i.e., APTES-modified glass substrate) fixed at an angle of 30°. A capillary-held GO meniscus between the confined geometry was forced to a reciprocating motion linearly by a motor-driven translation stage at a constant speed of 5 mm s^−1^ and deposition number of 50 cycles to deposit the GO sheet on the APTES-modified glass substrate. In this process, carboxyl groups in the GO basal plane were bonded covalently with the terminal groups of APTES. The resulting GO thin films deposited on the glass substrates were reduced thermally at 200 ℃ for 8 h to enhance the APTES-GO bond (i.e., OC=NH bond) and dissociate the oxygen functional groups of the GO domain. Conventional photolithography was used to pattern the thermally rGO thin film by spin-coating AZ-GXR 601(AZ Electronic Materials Co., Wiesbaden, Germany), a type of PR, on the rGO thin film at 3500 rpm for 40 s. Soft baking was progressed on a hot plate at 110 °C for 90 s to produce a robust PR layer by evaporating the solvent in PR. The UV exposure process (power of 20 mW cm^−2^ for 5 s) was performed using a customized photomask by considering the characteristics of AZ-GXR 601, a positive PR that softens in response to light. Subsequently, the solvent in PR was evaporated again by hard baking (at 110 °C for 90 s) to enhance the hardness of the PR and adhesion from the rGO surface. As the final development process proceeded using AZ-300MIF (Merck, Darmstadt, Germany) for 60 s, the desired photoresist-based line patterns were constructed on the rGO film. A mild O_2_ plasma etching process (100 W, 100 sccm, 7 min) was then introduced to remove the rGO region (i.e., uncovered PR pattern). Finally, the rGO-based patterned substrate was fabricated with the topographically/chemically defined region.

### Characterization of the rGO-based patterned substrate

The morphology of the line-patterned rGO film was observed by optical microscopy (Olympus BX51) in reflective mode and atomic force microscopy (Park Systems, NX-10) in non-contact mode. The localized charge and surface potential distribution of the sample surface (i.e., rGO and glass surface) were analyzed by KPFM (Kelvin probe Force microscopy) with a size of 20 × 20 µm^2^ using an NCSTAu cantilever. The thermally rGO films were evaluated by Raman spectroscopy with 532 nm laser excitation (UniNanoTech, UniRam-II) over the 500–3500 nm wavelength. The chemical composition of the rGO surface and silanol-terminated glass surface was determined by X-ray photoelectron spectroscopy (XPS, Kratos Analytical, AXIS SUPRA) with an Al-Kα excitation source (1486.6 eV). Using the C 1s peak for graphitic carbon (BE = 284.8 eV) as a reference, the measured binding energy was calibrated with regard to the charge shift. Optical transmittance measurements were performed using a UV–vis spectrophotometer (Thermo Scientific, Evolution 22) in the 400–800 nm wavelength.

### Cell culture

Tonsil-derived MSCs were provided by the Department of Otorhinolaryngology-Head and Neck Surgery, Pusan National University Hospital. The MSCs were isolated from the human tonsil tissue of a patient, as described previously [72, 73]. MSCs were cultured in α-minimum essential medium supplemented with 10% fetal bovine serum in a humidified atmosphere containing 5% CO_2_ in an incubator at 37 °C. The subculture was performed when the cell density reached the 70–80% area of the plate. Briefly, the cells were washed twice with Hank’s balanced salts solution and detached from the dishes by a treatment with 0.05% trypsin-EDTA for 3–5 min. The dissociated cells were collected into a conical tube, centrifuged for 4 min at 1000 rpm, and the cell pellets were resuspended in a cell culture medium. To seed the cells, the rGO and glass were placed on a 12 well plate and UV sterilized for at least 12 h. The resuspended cells were seeded at 2 × 10^4^ cells/well. To induce the differentiation of MSCs to SMCs, the MSCs were serum-starved in α-minimum essential medium free media for 24 h, followed by a treatment with TGF-β1 2 ng ml^−1^ for four days (refer to the scheme of Additional file 1: Fig. S15) [74].

### Proliferation assay

The proliferation ability of the cells on rGO and glass was assessed using an MTT assay (10× MTT (-[4,5-dimethylthiazole-2-yl]-2,5-diphenyltetrazolium bromide) diluted to 1× MTT solution with α-minimum essential medium). The cell growth media were aspirated and washed with Hank’s balanced salts solution, and the 1× MTT solution was added to the 400 µl/well. After forming formazan crystals in the cell culture incubator for 2 h, the 1× MTT solution was removed, and dimethyl sulfoxide (Sigma, USA) was added at 400 µl/well. Formazan dissolved in dimethyl sulfoxide was pipetted into a 96 well plate, and the absorbance was measured at 560 nm using a SunriseTM microplate reader (TECAN, Switzerland).

### Western blotting

To extract the protein from cells, the cells were lysed in a buffer (20 mM Tris-HCl, 1 mM EGTA, 1 mM EDTA, 10 mM NaCl, 0.1 mM phenylmethylsulfonyl fluoride, 1 mM Na_3_VO_4_, 30 mM sodium pyrophosphate, 25 mM β-glycerol phosphate, and 1% Triton X-100, pH 7.4). The cell lysates were sonicated and centrifuged at 12,000 rpm at 4 °C for 10 min, and the supernatants except for the pellet were used for western blotting. The lysates were resolved by sodium dodecyl sulfate-polyacrylamide gel electrophoresis and transferred onto nitrocellulose membranes. The membranes were blocked with 5% non-fat milk blocking buffer for 1 h and incubated overnight with the primary antibodies (α-SMA, Calponin, SM22α, Vimentin, Desmin, PDGFRβ, CX43, N-cadherin, and GAPDH). The attached secondary antibodies, which were conjugated to horseradish peroxidase for chemiluminescence, were incubated for 1 h and detected using an ECL kit (Amersham Biosciences, GE Healthcare, USA).

### Cell imaging

For immunocytochemistry analysis, the cells were fixed with 4% PFA for 30 min, washed, and permeabilized with PBS containing 0.1% Triton-X for 10 min. The cells were blocked with 5% bovine serum albumin blocking buffer for 1 h and incubated overnight with the primary antibodies (β-tubulin, Phalloidin, calponin, and SM22α) at 4 °C, followed by incubation with DAPI solution (Sigma, USA) and the secondary antibodies conjugated with Alexa-fluorescent dyes at 4 °C for 2 h. The cells were mounted on a slide glass with a Prolong gold antifade reagent (Thermofisher, USA). Images were obtained by confocal microscopy (LSM800, Zeiss, Germany). Optical microscopy was performed using an EVOS M5000 (Thermofisher, USA). Continuous time width imaging of live cells was performed with the support of EVOS Onstage Incubator.

### Cell morphology analysis

The cell length and arrangement angle were measured using the ImageJ program. The aspect ratio was calculated by dividing the length of the minor axis by the major axis. Graphing of the cell orientation was processed using the Origin program.

### Flow cytometry analysis

The cultured cells were harvested by trypsin-EDTA treatment, and the suspended single cells were fixed with 4% paraformaldehyde for 30 min. After fixation, the cells were washed with PBS and permeabilized with 1% Triton-X-100 in PBS for 30 min, and sequentially, blocked with 5% bovine serum albumin buffer for 1 h. Then, the cells were stained with primary antibodies (SM22α, calponin) at 4 °C for overnight and incubated with fluorescent dye conjugated-secondary antibodies at 4 °C for 2 h. The measurement was performed by using Attune NxT (Thermofisher, USA) and analyzed with FlowJo (ver10, Tree Star Inc.).

### Collagen gel contraction assay

This assay was performed by following the previously report [32]. Briefly, the cells were trypsinized and resuspended at 1 × 10^6^ cells ml^−1^ in α-MEM. Rat tail collagen type I (corning, USA) was chilled and titrated to pH 7.4 with 1 N NaOH. The cells were added to collagen gel solution to achieve a final concentration of 3 mg collagen/ml and 4 × 10^5^ cells/ml. The cell/collagen mixtures were added to a 24-well plate at 500 µl/well and polymerize for 1 h at 37 °C. After gelation, collagen gel lattices were mechanically released from the bottom of the tissue culture dishes by gently pipetting medium at the lattice-dish interface to initiate collagen gel contraction. The extent of contraction of each collagen gel lattices was analyzed by measuring dimension of the lattice before release and at 2 h after release using a digital CCD camera. The area of gel lattices was measured and analyzed using the Scion Image software.

## Supplementary Information

**Additional file 1: Figure S1.** Raman spectra on rGO and glass area. **Figure S2.** Optical transmittance of gradient rGO stripe pattern and rGO film. **Figure S3.** XPS spectra of C1s (a) and N1s (b) of the APTES-modified glass substrate. **Figure S4.** (**a**) Optical micrographs of MSCs on glass and rGO surface (**b**) Proliferation of MSCs on the tissue culture plate, glass, and rGO substrate. **Figure S5.** The magnified single-line pattern of MSCs depending on rGO/glass pattern spacing. **Figure S6.** Time-lapse image in recording MSCs movement on the 100 μm pattern spacing of glass/rGO. **Figure S7.** AFM image and height profiles of the micropatterned glass/rGO substrate. **Figure S8.** Potential distribution mapping for the surface charge at the glass/rGO region. **Figure S9.** Cell-to-Cell interaction between the aligned MSCs on glass/rGO patterned substrate. **Figure S10.** MSCs behavior on the unpatterned glass substrate. **Figure S11.** Real-time observations for the migration of MSCs cultured on 40 μm pattern spacing of glass/rGO. **Figure S12.** Histogram of the angular orientation of MSCs distributed by the glass/rGO pattern spacing formed on the cell substrate. **Figure S13.** Captured micrographs from time-lapse observations in recording MSCs migration on the cross-patterned glass surrounded by rGO. **Figure S14.** Cytoskeletal arrangement of MSCs cultured on the cross-patterned glass surrounded by rGO. **Figure S15.** The culture protocol to induce the quiescence of MSCs and TGF-β1-induced differentiation into SMCs. **Figure S16.** Quiescent MSCs cultured on 40 μm pattern spacing of rGO/glass. **Figure S17.** Angular orientation of quiescent MSCs distributed by the glass/rGO pattern spacing. **Figure S18**. TGF-β1-induced differentiation of MSCs to SMCs on the gradient patterned rGO/glass substrate. **Figure S19.** Flow cytometry analysis of SMC-specific markers for quiescent MSCs cultured on the pattern spacing of 100 μm; the peaks demonstrate direct comparison to TGF-β1-induced SMCs. **Figure S20.** (a) Representative optical micrographs of contractile collagen gel. (b) The measured result on the reduction of collagen gel surface area; the data shown as the mean ± SD (n=3). *P<0.05 **Figure S21.** YAP/TAZ expression of quiescent MSCs cultured on an anisotropic rGO/glass patterned substrate. **Table S1.** List of antibodies used in the present study. WB: western blotting/ICC: immunocytochemistry. **Table S2.** Peak area ratios of the oxygen-containing group to the C-C bond obtained by XPS.

**Additional file 2: Movie S1.**

**Additional file 3: Movie S2.**

**Additional file 4: Movie S3.**
