# Supplementary material for: Phenotypic change of mesenchymal stem cells into smooth muscle cells regulated by dynamic cell-surface interactions on patterned arrays of ultrathin graphene oxide substrates
Source: J Nanobiotechnology. 2022 Jan 4;20:17. doi: 10.1186/s12951-021-01225-4 (PMC8725258; doi:10.1186/s12951-021-01225-4)
Supplement: Supplementary file 1 — Additional file 1: Figure S1. Raman spectra on rGO and glass area. Figure S2. Optical transmittance of gradient rGO stripe pattern and rGO film. Figure S3. XPS spectra of C1s (a) and N1s (b) of the APTES-modified glass substrate. Figure S4. (a) Optical micrographs of MSCs on glass and rGO surface (b) Proliferation of MSCs on the tissue culture plate, glass, and rGO substrate. Figure S5. The magnified single-line pattern of MSCs depending on rGO/glass pattern spacing. Figure S6. Time-lapse image in recording MSCs movement on the 100 μm pattern spacing of glass/rGO. Figure S7. AFM image and height profiles of the micropatterned glass/rGO substrate. Figure S8. Potential distribution mapping for the surface charge at the glass/rGO region. Figure S9. Cell-to-Cell interaction between the aligned MSCs on glass/rGO patterned substrate. Figure S10. MSCs behavior on the unpatterned glass substrate. Figure S11. Real-time observations for the migration of MSCs cultured on 40 μm pattern spacing of glass/rGO. Figure S12. Histogram of the angular orientation of MSCs distributed by the glass/rGO pattern spacing formed on the cell substrate. Figure S13. Captured micrographs from time-lapse observations in recording MSCs migration on the cross-patterned glass surrounded by rGO. Figure S14. Cytoskeletal arrangement of MSCs cultured on the cross-patterned glass surrounded by rGO. Figure S15. The culture protocol to induce the quiescence of MSCs and TGF-β1-induced differentiation into SMCs. Figure S16. Quiescent MSCs cultured on 40 μm pattern spacing of rGO/glass. Figure S17. Angular orientation of quiescent MSCs distributed by the glass/rGO pattern spacing. Figure S18. TGF-β1-induced differentiation of MSCs to SMCs on the gradient patterned rGO/glass substrate. Figure S19. Flow cytometry analysis of SMC-specific markers for quiescent MSCs cultured on the pattern spacing of 100 μm; the peaks demonstrate direct comparison to TGF-β1-induced SMCs. Figure S20. (a) Representative optical mic [file 12951_2021_1225_MOESM1_ESM.docx]

**Supplementary Information**

Phenotypic Change in Mesenchymal Stem Cells to Smooth Muscle Cells Guided by the Cell-Surface Interaction on Patterned-Arrays of Ultrathin Graphene Oxide Films

*Rowoon Park*^a,1^, *Jung Won Yoon*^b,1^, *Jin-Ho Lee*^c^, *Suck Won Hong*^a,*^, *and Jae Ho Kim*^b,*^

^a^ Department of Cogno-Mechatronics Engineering, Pusan National University, Busan 46241, Republic of Korea

^b^ Department of Physiology, School of Medicine, Pusan National University, Yangsan, 50612, Republic of Korea

^c^ Department of Biomedical Convergence Engineering, Pusan National University, Yangsan 50612, Republic of Korea

^1^ These authors contributed equally to this work.

*****Corresponding authors: E-mail: [swhong@pusan.ac.kr](mailto:swhong@pusan.ac.kr) and jhkimst@pusan.ac.kr

KEYWORDS: stem cells, self-assembly, lithography, tissue engineering, smooth muscle cells

**Table S1.** List of antibodies used in the present study. WB: western blotting/ICC: immunocytochemistry

| Antibody | Catalog | Application | Dilution ratio |
| --- | --- | --- | --- |
| α-SMA | ab5694, Abcam | WB | 1:1000 |
| Calponin | C2687, Sigma Aldrich | WB | 1:1000 |
|  |  | ICC | 1:500 |
| SM22α | ab14106, Abcam | WB | 1:1000 |
|  |  | ICC | 1:500 |
| Vimentin | 5741, Cell signaling | WB | 1:1000 |
| Desmin | ab15200, Abcam | WB | 1:1000 |
| PDGFRβ | sc432, Santacruz | WB | 1:500 |
| CX43 | ab11370, Abcam | WB | 1:1000 |
| N-cadherin | 610920, BD Biosciences | WB | 1:1000 |
| YAP | 4912, Cell signaling | WB | 1:1000 |
| TAZ | 4883, Cell signaling | WB | 1:1000 |
| GAPDH | sc47724, Santacruz | WB | 1:1000 |
| Alexa flour 568 Phalloidin | A12380, Thermofisher | ICC | 1:500 |
| Alexa flour 488 goat anti-mouse | A11029, Thermofisher | ICC | 1:200 |
| Alexa flour 568 goat anti-rabbit | A11011, Thermofisher | ICC | 1:200 |
| goat anti-mouse IgG-HRP | A17168, Thermofisher | WB | 1:5000 |
| Donkey anti-Rabbit Ig-HRP | ab6802, Abcam | WB | 1:5000 |


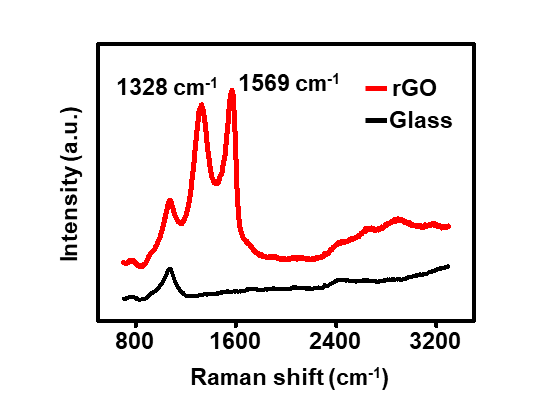


**Figure S1.** Raman spectra on rGO area (red) and glass area (black).


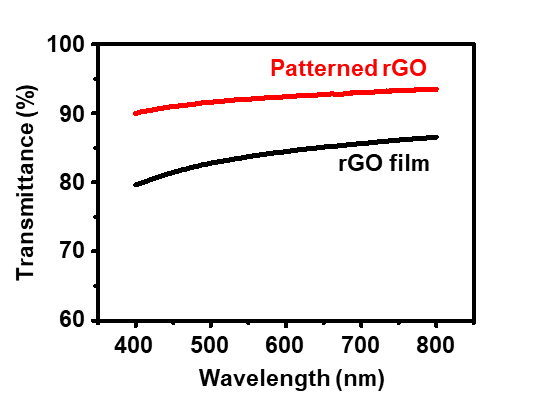


**Figure S2.** Optical transmittance of gradient rGO stripe pattern and rGO film.


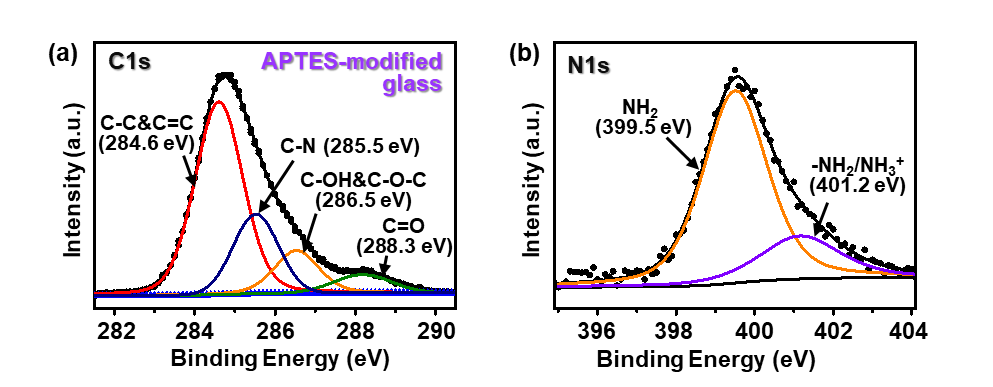


**Figure S3.** X-ray photoelectron spectroscopy (XPS) spectra of C1s (a) and N1s (b) of the APTES-modified glass substrate.

|  | **C-OH&C-O-C/C-C** | **C=O/C-C** | **C-N/C-C** |
| --- | --- | --- | --- |
| **rGO region** | **0.64** | **1.09** | **0.72** |
| **Glass region** | **1.26** | **0.79** | **0.60** |
| **APTES-glass** | **0.45** | **0.43** | **0.52** |

**Table S2.** Peak area ratios of the oxygen-containing group to the C-C bond obtained by XPS.


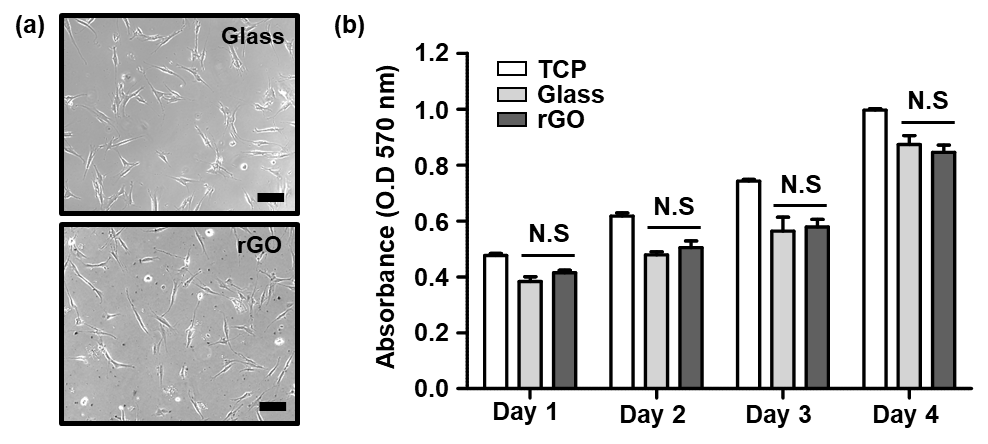


**Figure S4.** (a) Optical micrographs of MSCs on glass and rGO surface, scale bar: 200 μm. (b) Proliferation of MSCs on the tissue culture plate (TCP), glass, and rGO substrate. The MTT assay was used for the evaluation, which indicated a significant level of proliferation compared to TCP at ≤ 0.05. Data are shown as the mean ± SD (n=3). NS = not significant.


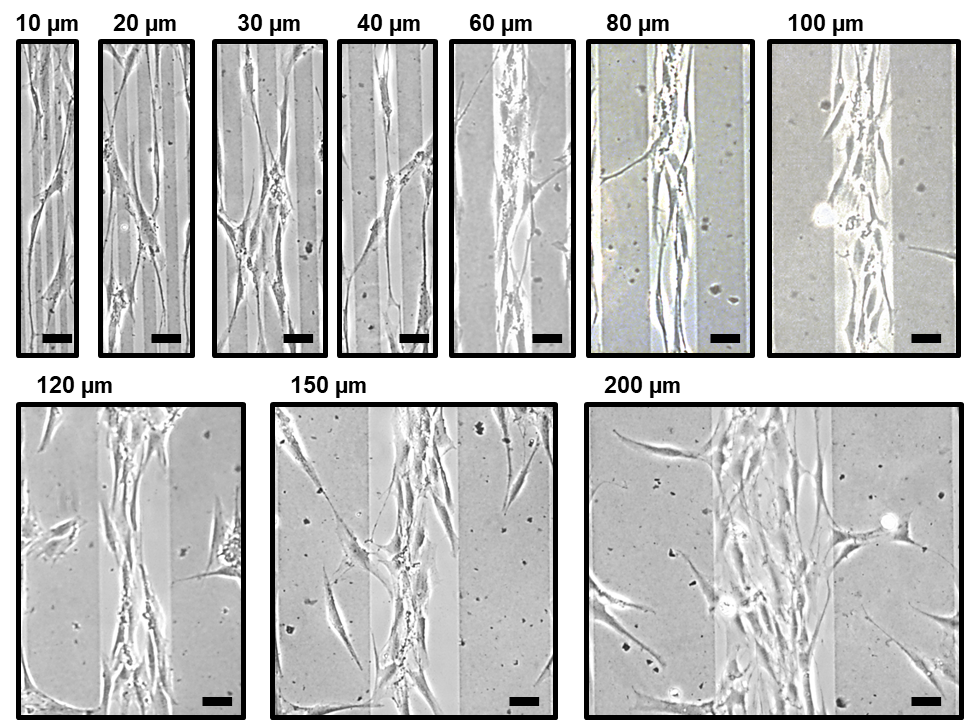


**Figure S5.** The magnified single-line pattern of MSCs depending on rGO/glass pattern spacing; which clearly revealed the cell-repellant template of rGO, scale bars: 50 µm.


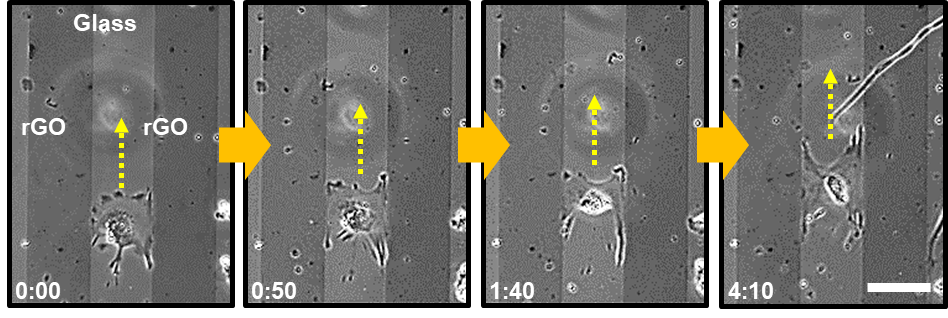


**Figure S6.** Captured images from the time-lapse movie in recording MSCs movement on the 100 μm pattern spacing of glass/rGO; captured time intervals (min/sec): 0:00/0:50/1:40/4:10, scale bar: 100 µm.


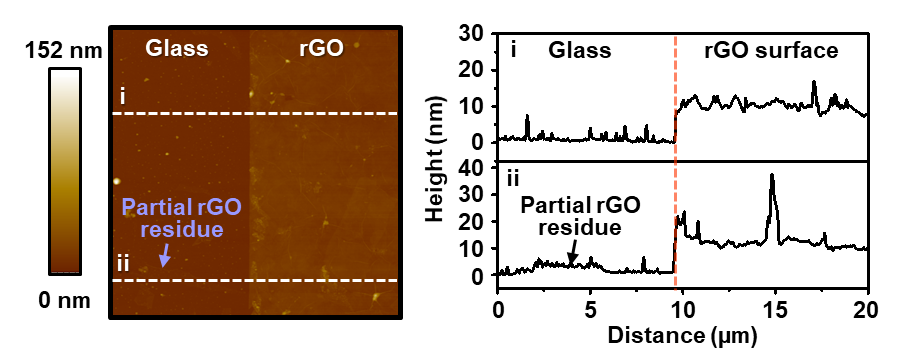


**Figure S7.** Topographic atomic force microscopy image and height profiles of the micropatterned glass/rGO substrate measured at a size of 20 × 20 µm^2^; the marked by white dotted lines (i and ii) were measured for the height profiles between the glass and rGO stripe.


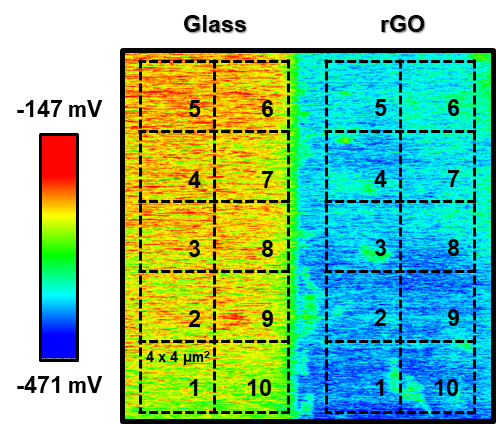


**Figure S8.** Potential distribution mapping for the surface charge at each glass and rGO region; the mean charge potential was extracted at a size of 4 × 4 µm^2^.


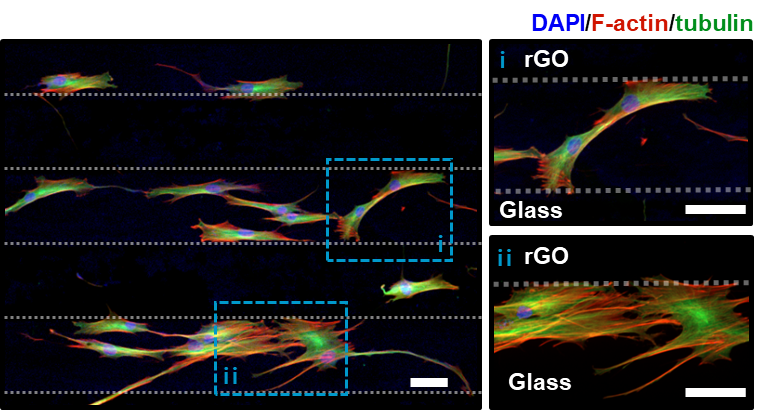


**Figure S9.** Cell-to-Cell interaction between the aligned MSCs on glass/rGO patterned substrate. Immunocytochemistry fluorescent micrographs represent the expression of F-actin (stress fiber, red), tubulin (microtubule, green), and DAPI (nuclei, blue) in the MSCs cultured on 100 μm pattern-spacing of glass/rGO, scale bar is sequentially 100, 50, and 50 µm.


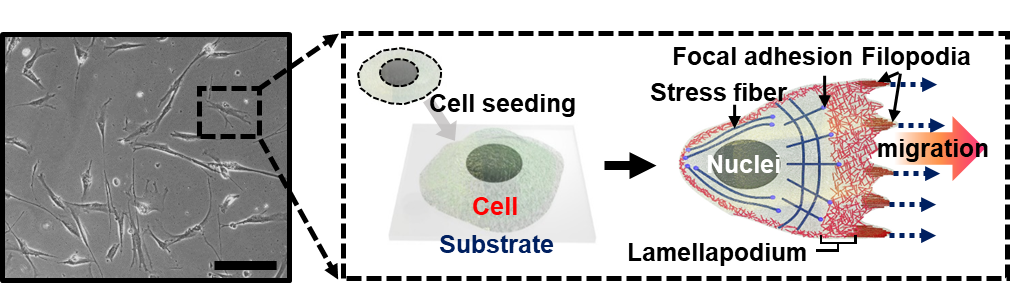


**Figure S10.** MSCs behavior on the unpatterned glass substrate, and schematic diagrams of MSCs adhesion and migration, scale bar: 200 μm.


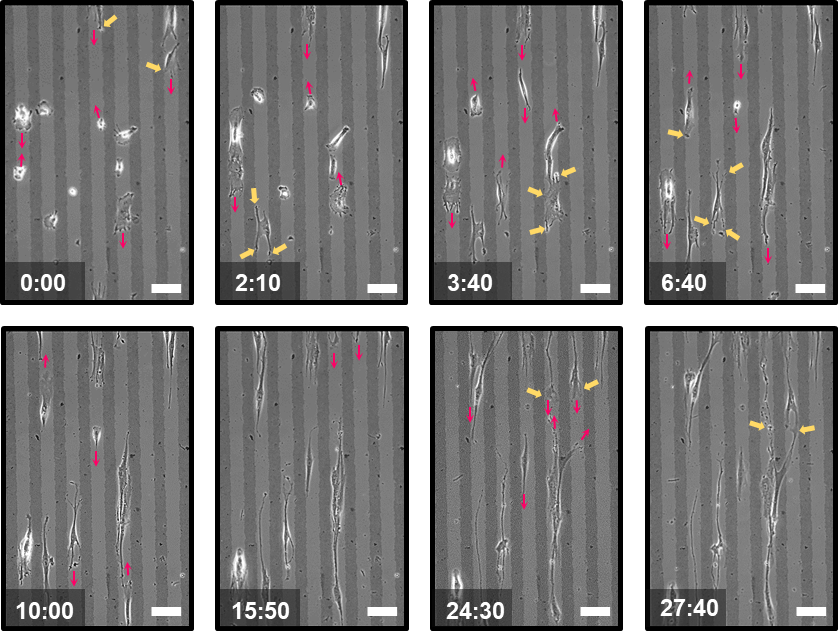


**Figure S11.** Real-time observations for the migration of MSCs cultured on 40 μm pattern spacing of glass/rGO. The images were captured from a time-lapse recording movie (time intervals (min/sec): 0:00/2:10/3:40/13:10/6:40/10:00/15:50/ 24:30/27:40), scale bars: 100 μm.


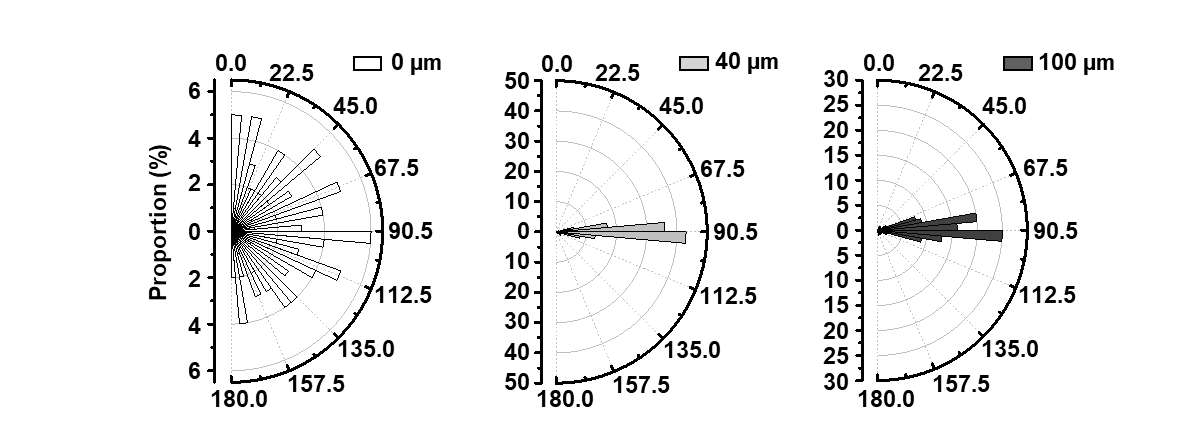


**Figure S12.** Histogram of the angular orientation of MSCs distributed by the glass/rGO pattern spacing formed on the cell substrate; approximately 90–120 cells were used in this observation.


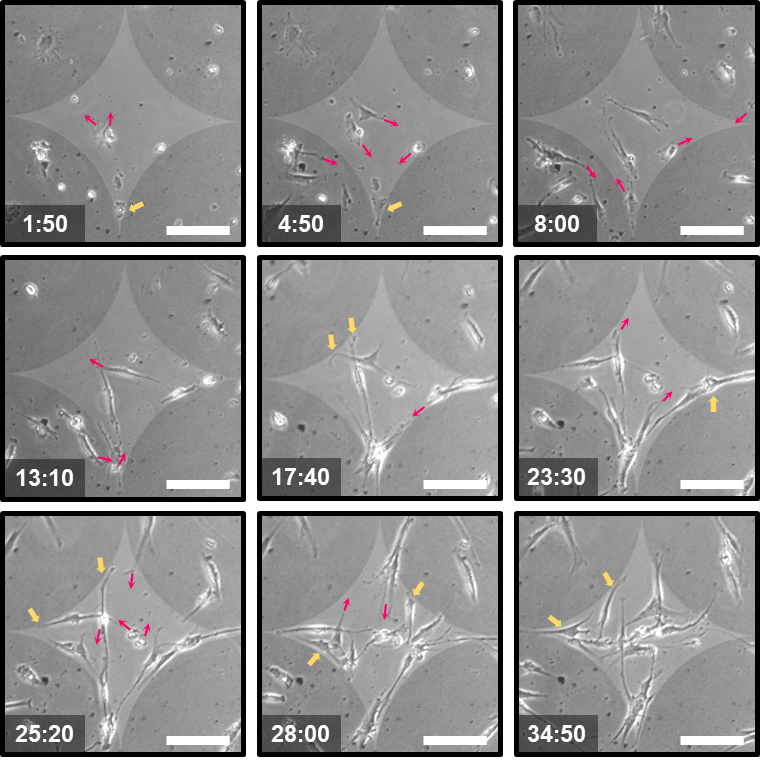


**Figure S13.** Captured micrographs from time-lapse observations in recording MSCs migration on the cross-patterned glass surrounded by rGO; Time-lapse interval (min:sec), 1:50/4:50/8:00/13:10/17:40/23:30 /25:20/ 28:00/34:50), scale bars: 200 μm.


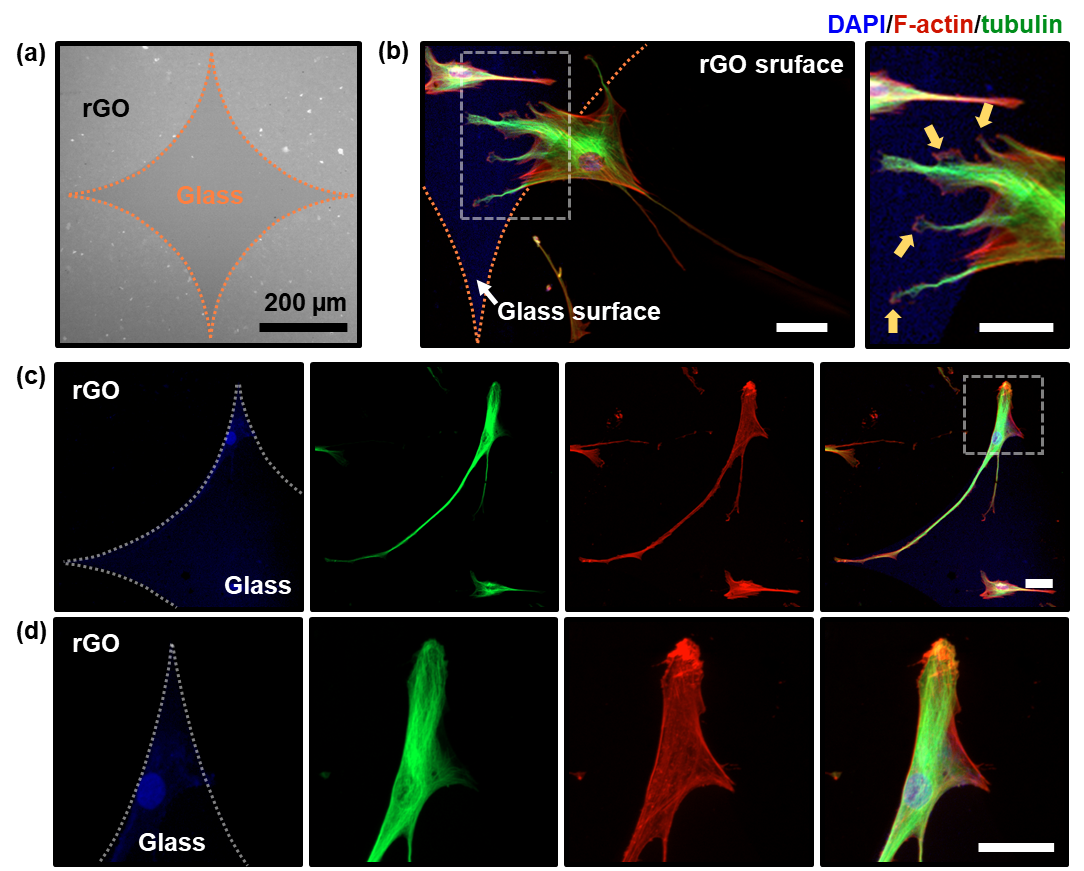


**Figure S14.** Cytoskeletal arrangement of MSCs cultured on the cross-patterned glass surrounded by rGO. (a) Optical micrograph of cross-patterned glass surrounded by the rGO surface, scale bar: 200 μm. (b-d) Zoom-in fluorescence micrographs for F-actin (stress fiber, red), tubulin (microtubule, green), and DAPI (nuclei, blue) in MSCs, the scale bars from top to bottom are 100, 30, 100, and 50 μm.


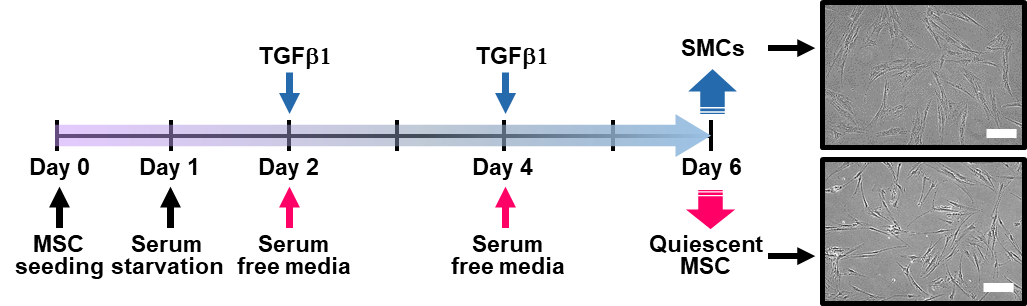


**Figure S15.** Schematic illustrations of the culture protocol to induce the quiescence of MSCs and TGF-β1-induced differentiation into SMCs, scale bars: 200 μm.


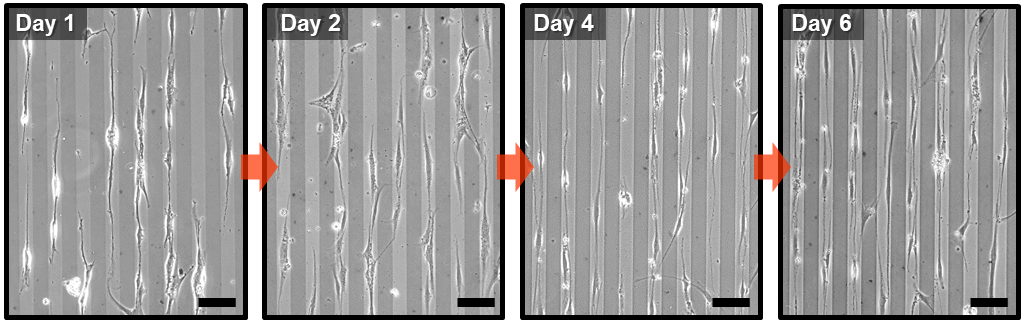


**Figure S16.** Quiescent MSCs cultured on 40 μm pattern spacing of rGO/glass. Optical micrographs of quiescent MSCs cultured in serum-free media at days 1, 2, 4, and 6 (scale bars: 100 μm).


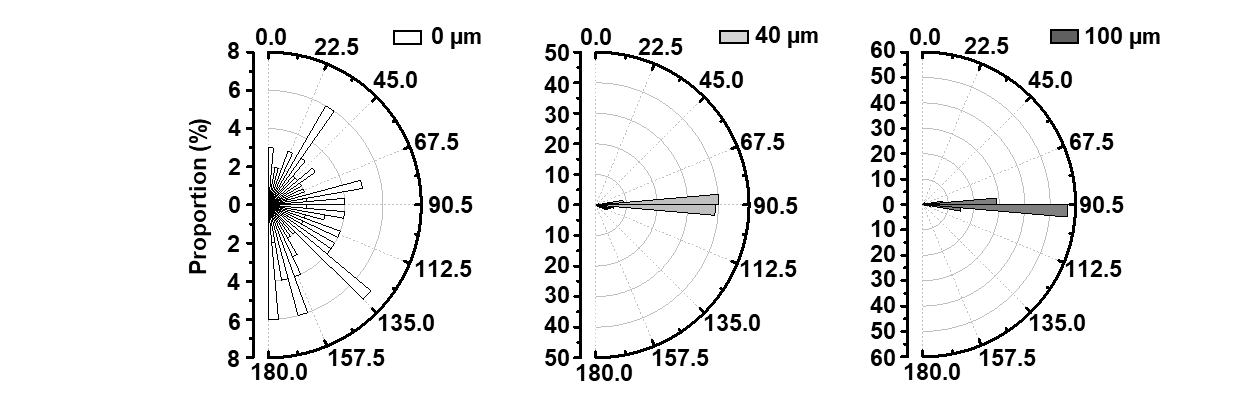


**Figure S17.** Angular orientation of quiescent MSCs distributed by the glass/rGO pattern spacing; approximately 90–120 cells were used in this observation.


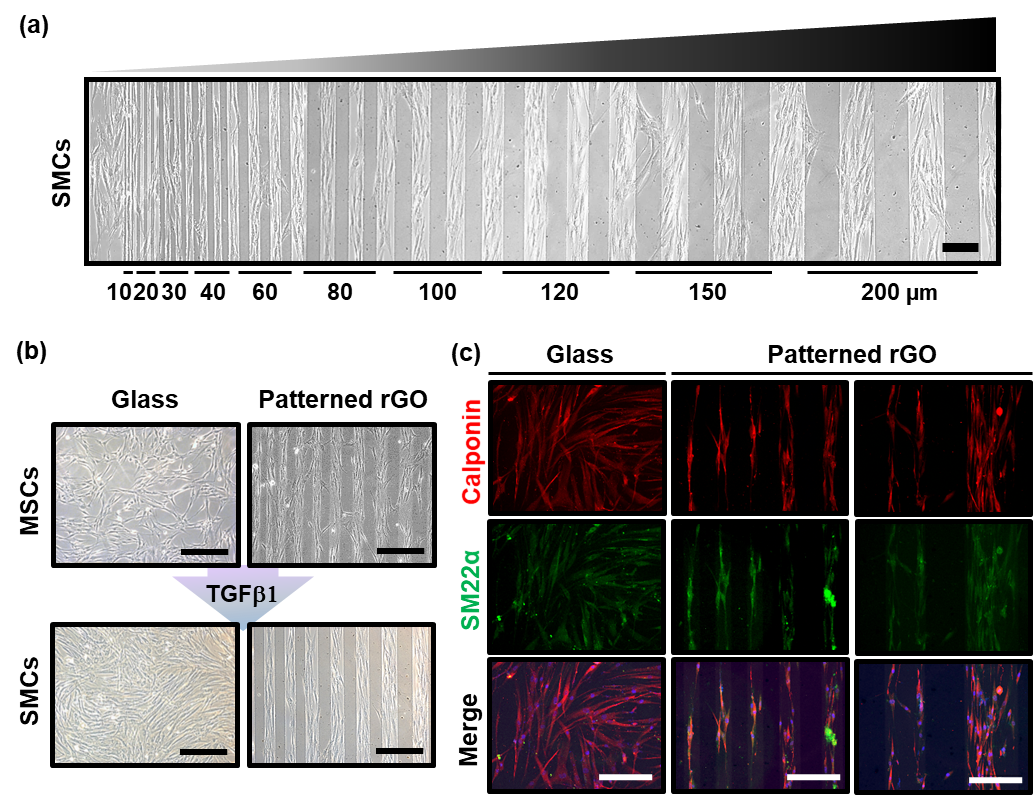


**Figure S18**. TGF-β1-induced differentiation of MSCs to SMCs on the gradient patterned rGO/glass substrate. (a) Morphological features of the SMCs differentiated from MSCs by a treatment with TGF-β1 on the gradient patterned rGO/glass substrate, scale bar: 200 µm. MSCs were seeded and treated with TGF- β1 (i.e., 10 ng ml-1) for four days. (b) Representative micrographs for the serum-starved MSCs and the SMCs, scale bars: 400 µm. (c) Immunocytochemistry fluorescent micrographs of the expression of calponin (red), SM22α (green), and DAPI (nuclei, blue) in SMCs cultured on a patterned rGO/glass substrate, scale bars: 200 µm.


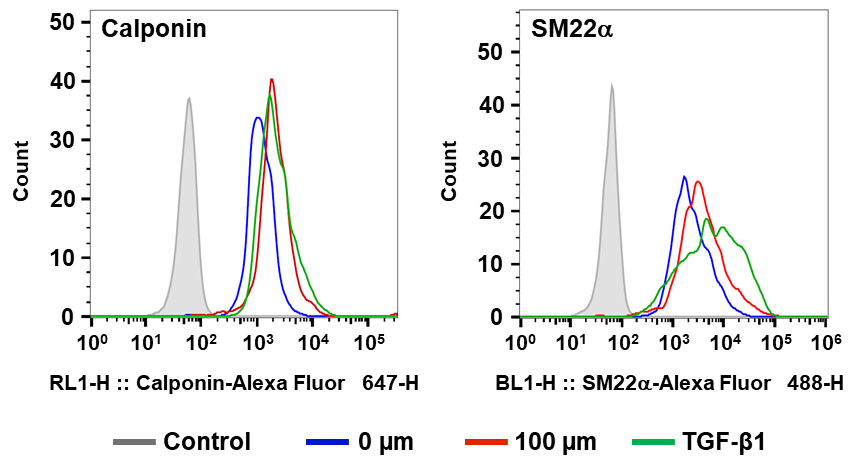


**Figure S19.** Flow cytometry analysis of SMC-specific markers (calponin and SM22α) in MSCs cultured at the pattern spacings of 0 μm or 100 μm, and the TGF-β1-treated MSCs.


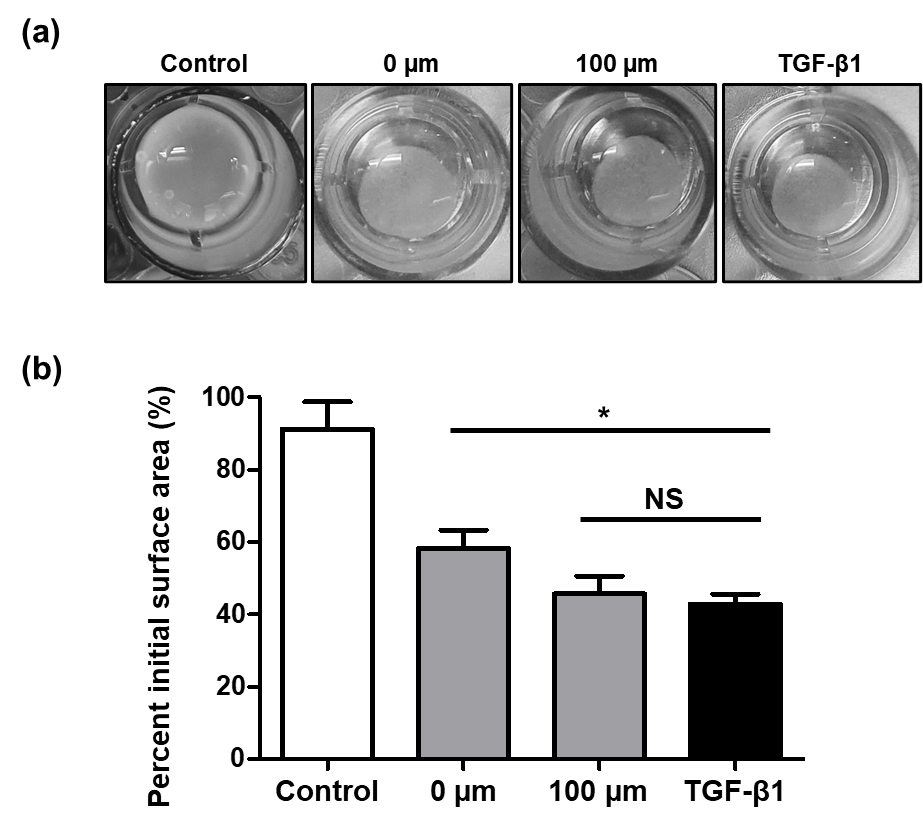


**Figure S20.** (a) Representative optical micrographs of contractile collagen gel. MSC (control), TGF-β1-induced SMCs, and MSC cultured at pattern spacings of 0 µm and 100 µm were embedded in collagen gel lattices, and gel contraction was photographed after 24 h by using a digital camera. (b) The area of the gel lattices was quantified with Scion image software, and the relative lattice area was obtained by dividing the area by the initial are of the lattice. The data shown as the mean ± SD (n=3). *P<0.05


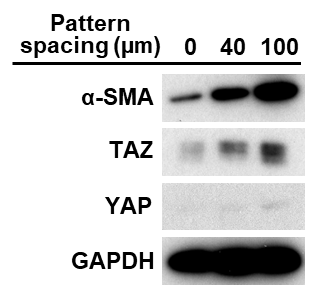


**Figure S21.** YAP/TAZ expression of quiescent MSCs cultured on an anisotropic rGO/glass patterned substrate. Western blot images of quiescent MSCs, representing the expression of α-SMA (SMC marker) and YAP/TAZ (Hippo pathway marker).
